# Supplementary material for: Magnitude and associated factors of sexual violence among female housemaids attending night school in Bahir Dar City, Northwest Ethiopia: Institution-Based Cross-Sectional Study, 2022
Source: PLoS One. 2024 Jul 25;19(7):e0307308. doi: 10.1371/journal.pone.0307308 (PMC11271934; doi:10.1371/journal.pone.0307308)
Supplement: Attachment — Submitted filename: PONE-D-23-31943 (1) Commented.pdf [file pone.0307308.s003.pdf]

PLOS ONE

Prevalence and associated factors of suicidal behavior among adolescents at Bahir Dar city, North West Ethiopia, community based study, 2021

--Manuscript Draft--

**Manuscript Number:**	PONE-D-23-31943
**Article Type:**	Research Article
**Full Title:**	Prevalence and associated factors of suicidal behavior among adolescents at Bahir Dar city, North West Ethiopia, community based study, 2021
**Short Title:**	Prevalence and associated factors of suicidal behavior among adolescents.
**Corresponding Author:**	Abdulbasit Sherfa, MPH<br>Werabe University<br>Werabe, ETHIOPIA
**Keywords:**	adolescents; Bahir Dar; Community; Ethiopia; Suicidal behavior
**Abstract:**	Background<br><br>Suicide is the second leading cause of death among young people aged 15 to 29 years old, according to the World Health Organization. However, it is a neglected public health issue, particularly in middle- and low-income countries such as Ethiopia. Despite the high prevalence of suicide and other harmful behaviour among this group in our country, limited evidence on suicidal behaviour and associated factors has been conducted among teenagers, particularly in community settings. As a result, the goal of this study was to look at the incidence and drivers of suicide conduct among adolescents in Bahir Dar.<br><br>Methods<br><br>A community-based cross-sectional survey was conducted among 692 teenagers in Bahir Dar, Northwest Ethiopia, from May 1 to 30, 2021. Face-to-face interviews with semi-structured and standardized questionnaires were used to collect data. For processing and analysis, the acquired data was entered into Epi-Data version 3.1 and exported to Statistical Package for Social Science version 24. To determine the independent components related with suicidal behaviour, bi-variable and multivariable logistic regression were use.<br><br>Results<br><br>The study included 631 teenagers, with a response rate of 91.18%. Suicidal behaviour was prevalent in 19.8% of the population (95% CI: 16.6, 22.8). When compared to their peers, adolescents with a family history of suicidal attempts had a 5.66 times (AOR = 5.66, CI = 2.44, 13.10) higher risk of suicidal behaviour. Similarly, adolescents who experienced stressful life events had a higher risk of suicide conduct (AOR = 2.91, CI = 1.45-4.91) than their peers. Adolescents who had negative childhood experiences exhibited a sevenfold (AOR = 7.11, CI = 3.56-14.20) higher risk of suicide behaviour than their peers. Adolescents who do not have a good relationship with their parents have a higher risk of developing suicidal behaviour (AOR = 5.59, CI = 2.59-12.08). Adolescents with significant interpersonal stress had around twice the chances [AOR = 2.17, 95% CI 1.07.4.40] of suicide behaviour as their peers. Adolescents who expressed more anger had 7.3 times the chances of reporting suicide conduct (AOR = 7.30, CI = 2.51-21.22) than those who expressed less anger. Suicidal activity was five times more likely in those who experienced depression [AOR = 5.32, CI = 2.64-10.70]. Adolescents with low social support were approximately 12.42 times [AOR = 12.42, CI = 5.95-25.95] more likely to report suicide behaviours than those with high social support.<br><br>Conclusion and Recommendation<br><br>The prevalence of suicidal behavior was high in this study showing a significant public health issue among adolescents that requires a great emphasis.

Powered by Editorial Manager® and ProduXion Manager® from Aries Systems Corporation

<table>
<tr><td>Order of Authors:</td><td>Belete Birhan
Michael Beka
Habte Belete, MSc
Abdulbasit Sherfa, MPH
Gidey Rtbey, MSc</td></tr>
<tr><td>Additional Information:</td><td></td></tr>
<tr><td>Question</td><td>Response</td></tr>
<tr><td>Financial Disclosure

Enter a financial disclosure statement that describes the sources of funding for the work included in this submission. Review the submission guidelines for detailed requirements. View published research articles from PLOS ONE for specific examples.

This statement is required for submission and will appear in the published article if the submission is accepted. Please make sure it is accurate.

Funded studies
Enter a statement with the following details:
• Initials of the authors who received each award
• Grant numbers awarded to each author
• The full name of each funder
• URL of each funder website
• Did the sponsors or funders play any role in the study design, data collection and analysis, decision to publish, or preparation of the manuscript?

Did you receive funding for this work?</td><td>The author(s) received no specific funding for this work.</td></tr>
<tr><td>Competing Interests

Use the instructions below to enter a competing interest statement for this submission. On behalf of all authors, disclose any competing interests that could be perceived to bias this work—acknowledging all financial support and any other relevant financial or non-financial competing interests.</td><td>The authors have declared that no competing interests exist.</td></tr>
</table>

<table>
<tr><td>This statement is required for submission and will appear in the published article if the submission is accepted. Please make sure it is accurate and that any funding sources listed in your Funding Information later in the submission form are also declared in your Financial Disclosure statement.

View published research articles from PLOS ONE for specific examples.

NO authors have competing interests

Enter: The authors have declared that no competing interests exist.

Authors with competing interests

Enter competing interest details beginning with this statement:

I have read the journal's policy and the authors of this manuscript have the following competing interests: [insert competing interests here]

* typeset</td><td></td></tr>
<tr><td>Ethics Statement

Enter an ethics statement for this submission. This statement is required if the study involved:

• Human participants
• Human specimens or tissue
• Vertebrate animals or cephalopods
• Vertebrate embryos or tissues
• Field research

Write "N/A" if the submission does not require an ethics statement.

General guidance is provided below. Consult the submission guidelines for detailed instructions. Make sure that all information entered here is included in the</td><td>Ethics approval and consent to participate
The study was approved by the Institutional review board (IRB) of Bahir Dar University, College of Medicine and Health Science [REF.178/2021]. Information about the study was explained to each study participant in the information sheet. Written informed consent was obtained from Adolescents aged 18 years and above (participants under the age of 18 were given verbal assent and written informed consent was obtained from their parents or guardians on their behalf).
The latter of ethical approval will be sent to PLOS ONE at a time when I get it from the first author on the way to revision of the manuscript. This is because the first author is by now living in part of the country in which internet is limited due to recent condition of the country, so I will upload it recently. Thank you.</td></tr>
</table>

**Methods section of the manuscript.**<br><br>**Format for specific study types**<br><br>**Human Subject Research (involving human participants and/or tissue)**<br>• Give the name of the institutional review board or ethics committee that approved the study<br>• Include the approval number and/or a statement indicating approval of this research<br>• Indicate the form of consent obtained (written/oral) or the reason that consent was not obtained (e.g. the data were analyzed anonymously)<br><br>**Animal Research (involving vertebrate animals, embryos or tissues)**<br>• Provide the name of the Institutional Animal Care and Use Committee (IACUC) or other relevant ethics board that reviewed the study protocol, and indicate whether they approved this research or granted a formal waiver of ethical approval<br>• Include an approval number if one was obtained<br>• If the study involved *non-human primates*, add *additional details* about animal welfare and steps taken to ameliorate suffering<br>• If anesthesia, euthanasia, or any kind of animal sacrifice is part of the study, include briefly which substances and/or methods were applied<br><br>**Field Research**<br><br>Include the following details if this study involves the collection of plant, animal, or other materials from a natural setting:<br>• Field permit number<br>• Name of the institution or relevant body that granted permission	
**Data Availability**<br><br>Authors are required to make all data underlying the findings described fully available, without restriction, and from the time of publication. PLOS allows rare exceptions to address legal and ethical concerns. See the PLOS Data Policy and FAQ for detailed information.	Yes - all data are fully available without restriction

A Data Availability Statement describing where the data can be found is required at submission. Your answers to this question constitute the Data Availability Statement and **will be published in the article**, if accepted.<br><br>**Important:** Stating 'data available on request from the author' is not sufficient. If your data are only available upon request, select 'No' for the first question and explain your exceptional situation in the text box.<br><br>Do the authors confirm that all data underlying the findings described in their manuscript are fully available without restriction?	
**Describe where the data may be found in full sentences. If you are copying our sample text, replace any instances of XXX with the appropriate details.**<br><br>• If the data are **held or will be held in a public repository**, include URLs, accession numbers or DOIs. If this information will only be available after acceptance, indicate this by ticking the box below. For example: *All XXX files are available from the XXX database (accession number(s) XXX, XXX.*).<br>• If the data are all contained **within the manuscript and/or Supporting Information files**, enter the following: *All relevant data are within the manuscript and its Supporting Information files.*<br>• If neither of these applies but you are able to provide **details of access elsewhere**, with or without limitations, please do so. For example:<br><br>*Data cannot be shared publicly because of [XXX]. Data are available from the XXX Institutional Data Access / Ethics Committee (contact via XXX) for researchers who meet the criteria for access to confidential data.*<br><br>*The data underlying the results*	The data underlying the results presented in the study are available from (include the name of the third party and contact information or URL).

presented in the study are available from (include the name of the third party and contact information or URL).<br>• This text is appropriate if the data are owned by a third party and authors do not have permission to share the data.<br><br>* typeset	
Additional data availability information:	Tick here if the URLs/accession numbers/DOIs will be available only after acceptance of the manuscript for publication so that we can ensure their inclusion before publication.

Manuscript Click here to access/download;Manuscript;Manuscript.docx

Prevalence and associated factors of suicidal behavior among adolescents at Bahir Dar city, North West Ethiopia, community based study, 2021

1 Belete Birhan, 2 Dr. Michael Beka, 2 Habte Belete, 3 Abdulbasit Sherfa, 4 Gidey Rtbey

College of medicine and health sciences, Bahir Dar University, Bahir Dar, Ethiopia

1, Department of Psychiatry, College of Medicine and Health Sciences, Wolayita Sodo University, Wolayita Sodo, Ethiopia

2, Department of Psychiatry, College of Medicine and Health Sciences, Bahir Dar University, Bahir Dar, Ethiopia

3, Department of Public Health, College of Medicine and Health Sciences, Werabe University, Werabe, Ethiopia

4, Department of Psychiatry, College of Medicine and Health Sciences, University of Gondar, Gondar, Ethiopia

Email addresses: beletebirhan@gmail.com, Michaelbeka27@yahoo.com, habte.belete@gmail.com, abdusherfa3@gmail.com, Gidur2006@gmail.com

***Correspondent Author:** Abdulbasit Sherfa, E-mail: abdusherfa3@gmail.com, Phone: +251-913-69-28-80*

ABSTRACT

Background: Suicide is the second leading cause of death among young people aged 15 to 29 years old, according to the World Health Organization. However, it is a neglected public health issue, particularly in middle- and low-income countries such as Ethiopia. Despite the high prevalence of suicide and other harmful behaviour among this group in our country, limited evidence on suicidal behaviour and associated factors has been conducted among teenagers, particularly in community settings. As a result, the goal of this study was to look at the incidence and drivers of suicide conduct among adolescents in Bahir Dar.

Methods: A community-based cross-sectional survey was conducted among 692 teenagers in Bahir Dar, Northwest Ethiopia, from May 1 to 30, 2021. Face-to-face interviews with semi-structured and standardized questionnaires were used to collect data. For processing and analysis, the acquired data was entered into Epi-Data version 3.1 and exported to Statistical Package for Social Science version 24. To determine the independent components related with suicidal behaviour, bi-variable and multivariable logistic regression were use.

Results: The study included 631 teenagers, with a response rate of 91.18%. Suicidal behaviour was prevalent in 19.8% of the population (95% CI: 16.6, 22.8). When compared to their peers, adolescents with a family history of suicidal attempts had a 5.66 times (AOR = 5.66, CI = 2.44, 13.10) higher risk of suicidal behaviour. Similarly, adolescents who experienced stressful life events had a higher risk of suicide conduct (AOR = 2.91, CI = 1.45-4.91) than their peers. Adolescents who had negative childhood experiences exhibited a sevenfold (AOR = 7.11, CI = 3.56-14.20) higher risk of suicide behaviour than their peers. Adolescents who do not have a good relationship with their parents have a higher risk of developing suicidal behaviour (AOR = 5.59, CI = 2.59-12.08). Adolescents with significant interpersonal stress had around twice the chances [AOR = 2.17, 95% CI 1.07.4.40] of suicide behaviour as their peers. Adolescents who expressed more anger had 7.3 times the chances of reporting suicide conduct (AOR = 7.30, CI = 2.51-21.22) than those who expressed less anger. Suicidal activity was five times more likely in those who experienced depression [AOR = 5.32, CI = 2.64-10.70]. Adolescents with low social support were approximately 12.42 times [AOR = 12.42, CI = 5.95-25.95] more likely to report suicide behaviours than those with high social support.

Conclusion and Recommendation: The prevalence of suicidal behavior was high in this study showing a significant public health issue among adolescents that requires a great emphasis.

Key words: Adolescents, Bahir Dar, Community, Ethiopia, Suicidal behavior

Introduction

Adolescence is a period of transition in the life of a human during which people obtain physiological and psychosocial maturity (1) . One in six people are aged 10-19 years. Multiple physical, emotional and societal changes, including exposure to poverty, abuse, or violence, can make adolescents prone to different mental health problems (2). it is a period of complex and an exceptional and developmental time, during which individuals can undertake different danger practices, including suicidal behavior, which covers suicidal ideation, suicide attempts and suicide itself (3) . Suicidal ideation denotes thoughts of engaging in behavior intended to end one's life, whereas suicide planning is defined as the preparation of a specific method through which one plans to die (4).

Suicidal behavior is among the leading cause of damage and death worldwide. It is a public health issue that is estimated to contribute more than 2.4% to the global burden of disease by the year 2020 (5).It is currently the 10th most common cause of mortality resulting in the loss of approximately one million lives every year (6). Globally there have been an estimated 804 000 suicide deaths arose in 2012, representing a twelve-monthly global age-standardized suicide rate of 11.4 per 100 000 population (15.0 for males and 8.0 for females). However, since suicide is a sensitive issue, and even illegal in some countries, it is very likely and under-reported. Every 40 seconds a person dies by suicide somewhere in the globe and many more attempt suicide. Suicides occur all over the world and throughout the entire life. Among young people 15-29 years of age, suicide is the second leading cause of death globally and represents a major nationwide health concern according to WHO (7, 8).Worldwide, about 4 million adolescents attempt suicide annually, resulting in at least 100,000 deaths (9). Suicide results for an estimated 6% of all deaths among young people globally (10). In low- and middle-income countries adolescent suicide account for 78% of all suicides (11).

In Africa, the overall magnitude of suicide ranged from 2–3% [range 0.7–6.0%] (12). Sub-Saharan Africa there are over 34,000 (IQR 13,141 to 63,757) suicides per year, with an overall incidence rate of 3.2 per 100,000 populations (13).Young people are self-harming and taking their own lives at alarming rates. According to WHO Global health estimates in 2015 around

67,148 adolescent deaths were reported, among those 11,920 adolescent aged 10-14 years and the rest 55,228 were aged 15-19 years (14) .

Suicide is a principal cause of death among adolescents. Self-harm is the most important risk factor for suicide, yet the majority of self-harm does not come to the attention of health services (15).Suicidal behavior, deliberate self-harm and non-suicidal self-injury are significant precursors of suicide in children and adolescent (16). It has been reported as a leading cause of psychiatric emergencies for adolescents and the strongest predictor of future suicide attempts and suicide death (17).

Suicidal thoughts and suicidal behavior's develop during adolescence and peak late in adolescence and early adulthood (18).Adolescent suicidal behavior is a neglected public health issue especially in middle and low-income countries including Ethiopia (19). it is a multipart and multidimensional phenomenon stemming from the interaction of several factors (20).

Worldwide, the possible risk factors of suicide include victimization (bullying, sexual harassment), poor psychological state related to depression, phobic disorders, anxiety, alcohol use disorder, child abuse, impulsivity, lack of parental understanding (1). Many studies have demonstrated that mental state problems, substance use, genetic and biological factors, poor physical health or physical disability, and family–environmental factors are associated with increased risk of suicidal behaviors in adolescents (6, 21). Different Evidence shows that adolescents of both genders who had suicide ideation and attempts are significantly more likely to commit suicide than those without suicidal ideation and attempts (22).

Reducing suicides is therefore a key public health target. Healthcare systems and services need to be strong and able to incorporate suicide prevention as a core component. Universal health coverage (UHC) ensures that the most vulnerable can access care. Communities play a critical role by addressing stigma and isolation, improving knowledge and awareness, and providing social support to vulnerable individuals. A tool kit to engage communities in suicide prevention has been made available by WHO (14).

In May 2013, the 66th World Health Assembly formally adopted the first-ever Mental Health Action Plan of the World Health Organization (WHO), Suicide prevention is an important

component of the Mental Health Action Plan, with the goal of reducing the rate of suicide in countries by 10% by 2020 (7).

The suicide rate, however, is rising most rapidly among young persons, particularly males 15 to 24 years of age, and the rate is still rising (12). Even though LMICs account for the bulk of suicides, high-income Western nations are the source of most of the knowledge about suicidal behaviours. Because there aren't many researches among teenagers in Ethiopia, little is known regarding the prevalence and determinants of suicide behaviour (suicidal thought, suicidal plan, and suicidal attempt). Therefore, the goal of this study was to evaluate the incidence of suicidal conduct in teenagers and its contributing factors on a local level.

Methods and Material

Study design and setting Area

We have conducted a community based cross-sectional study in Bahir Dar city Northwest Ethiopia which is 565 kilo meters from Addis Ababa. According to Bahir Dar city mayor office report in 2019/2020, the estimated population in the city was 37[illegible],073. From the total population the number of adolescent (between 10-19 years) was 84,382. The city has one comprehensive specialized hospital, one specialized public hospital, one primary hospital, and four private hospitals, which give service to the city and surrounding population. However, only the comprehensive specialized and specialized hospitals have inpatient psychiatric service. Study was carried out from May1-30, 2021.

Participants

The study was targeting on adolescent age (10-19 years old) residing in Bahir Dar city, North West Ethiopia. Participant's age (10-19) years residing Bahir Dar selected sub city for at least 6 month during study period were included and those participants who are in severe illness and couldn't communicate well for data collectors were not included.

Sample size, sampling procedure and data collection

Sample size was determined based on the single population proportion formula by using the following assumptions: $N = Z_{\alpha/2} \times P(1-P)/W^2$

By taking the proportion 22.5% (23) from previous study which was done in Ethiopia and 95% confidence interval was used, 4% margin of error sample size was calculated as:

Where $Z_{\alpha/2}$ = confidence level (1.96) at CI of 95%

N= Sample size

P=22.5% (proportion of adolescent who have suicidal ideation)

W= margin of sampling error.

1- P= sample error proportion of adolescent who have no suicidal ideation

N = (1.96)2 (0.225) (1-0.225)/ (0.04)2= 419 then multiply by 1.5 for design effect which gives 629. By assuming a 10% non-response rate, the final sample size was 692.

Multistage sampling technique was used to select the sub-cities (three) from the total of six and to select respective administrative kebeles (the smallest administrative unit) (six) from total of seventeen. The households in the administrative kebeles were selected by systematic random sampling technique after identifying an initial starting household by use of random number. Eligible adolescents in the selected household were further selected and interviewed. Only one adolescent member of the house hold was selected by lottery method for the interview towards suicide behavior. Data was collected by trained health extension workers and psychiatry nurses with interview by semi-structured questionnaire which was translated into Amharic version (local working language).

Measurements

Suicidal behavior of adolescent was assessed by using the short version of the mini-international neuropsychiatric interview (MINI).it consists of six items that are scored “yes or no” Among those items, 1–5, record whether an event has occurred during the last month, and item 6 records the lifetime occurrence of the event. If the participant response one "yes" answer from six item

(MINI) questions considered as risk for suicidal behavior. MINI has good validity, reliability, and reliability (chronbach's alpha = 0.84) (24). Social support assessed by using oslo-3 social support scale with sum of 3-14, that consist three items; poor(3-8), moderate (9-11) and strong(12-14)(25). Depression was assessed by a nine-item Patient Health Questionnaire-9, which has four response categories referring to the amount of time that the symptom was present (not at all (0), several days (1), more than half of the days (2), nearly every day (3)), with a total score ranging from 0 to 27 (26). For Assessment of adverse child hood experience adverse childhood experience (ACE) Questioner was used (27). self-esteem was assessed by using Rosenberg self-esteem scale people scoring between 15 and 25 is average. A score of less than 15 suggests low self-esteem and score greater than 25 suggests high self-esteem (28). Anger expression was assessed by using the Spiel Berger Anger-Out Expression Scale, which has 8-item; scale evaluates respondents' coping methods, particularly outward expressive behavior, when angry. Low anger expression (scores < 10), moderate anger expression; and high anger expression (scores ≥15) (29). Stressful life events of participants were assessed by SLESQ**.** The SLESQ self-report measure for non-treatment seeking samples that assesses lifetime exposure to traumatic events. Eleven specific and two general categories of events, such as a life-threatening accident, physical and sexual abuse, witness to another person being killed or assaulted (30). Interpersonal stress was asses by using Bergen Social Relationships Scale BSRS. The scale consists of six items. Individual high scores (above mean) of BSRS has high interpersonal stress (31).Clinical variables like, family history of suicidal attempt and other factors like growing up with their parent were assessed by asking the participants

Analysis

The collected data was entered into Epi-data version 3.1, exported to SPSS software version 24.0, and checked for inconsistencies and missing values by running frequencies and other data explorations. Inconsistencies and missing values was cleaned by checking the original questionnaire. Frequency distributions, mean, median and interquartile range (IQR) was computed.

Beyond descriptive statistics, associations between the dependent variables and the independent variables were analyzed by calculating the Odds Ratios and 95 % confidence interval. First,

Bivariate analysis was performed on each variable and a respective crude odds ratio (COR) was calculated. Independent variables with marginal associations ($P \leq 0.20$) in the bivariate analysis were entered in a multivariate logistic regression analysis in order to detect independent factors of suicidal behavior. The significant association of independent variables with the dependent variable was assessed by using 95% confidence interval and a respective adjusted odds ratio (AOR). A two tailed-sided p-value of ≤0.05 will be taken as statistically significant.

Ethical considerations

Ethical clearance was obtained from Institutional review board (IRB) of Bahir Dar University, College of Medicine and Health Science. Then letter of permission was obtained from Bahir Dar city administration health office and permission was granted from the city administrator. The aims of the study were explain for the study participants and data were collected after assent and written consent. Adolescents aged 18 years and above were gave informed written consent to participate. Participants under the age of 18 were verbal assent to the study and then written consent was obtained from their parents /guardians/ on their behalf. Both adolescents and parents were informed that they had the right to refuse to answer any question at any time. Referral was given for participants who had suicidal behavior and depressive symptoms.

Result

Socio demographic Characteristics of the Respondents

In this study, out of 692 individuals, 91.2% (631) responded to the suicidal behaviour interview. More than half of the respondents were in the middle adolescent age group, with a mean (SD) of 16.1 (2.0) years. Almost all (99.4%) of the respondents were unmarried. The majority (83.7%) of the respondents was living with their parents; 6.5% lived with someone other than their parents; and the rest (9.8%) lived alone. More than half (55.2%) of the participants were female (Table 1).

Psychosocial and Substance Related Factors

This study found that 13.5% of the participants have a family history of suicidal attempts, and 0.3% have a family history of committing suicide. Close to 80% of participants grew up with their biological parents. 14.1% (89) of respondents don't have a good relationship with their

family. Almost all participants (99.7% have good relationships with peers. Large proportions of respondents report moderate social support. The use of tobacco, alcohol, and chat was 4.4%, 9.2%, and 5.1%, respectively (see table 2).

Clinical and other related factors

From the total participants, 121 (19.2%) had depression. Concerning stressful life events, 40.9% report having stressful life experiences. More than one-third (36.3%) of the respondents report having had adverse childhood experiences. From the study participants, 24.6%, 60.4%, and 6% report having low, moderate, and high self-esteem, respectively. 62.4% and 13.8% of the study participants had moderate and higher anger expression behaviours, respectively. Around 60% of study participants had a fear of COVID-19, and 51.5% of participants had high interpersonal stress.

The magnitude of suicidal behavior

This study found the prevalence of suicidal behaviour to be 19.8% (125) with a 95% CI of 16.6-22.8. Out of which, almost half (48%) of them were in the late adolescent stage (18–19), and 71.2% (89/125) were female. 34% (42/125) had a family history of suicidal attempts, 85% (106/125) had adverse childhood experiences, 22.4% (28/125) had ever used alcohol, more than one-third of them had no good relationship with their parents (48/125), and 76% (95/125) had stressful life experiences. 53.6% (67/125) and 33.6% (42/125) had moderate and high anger expression behaviours, respectively. For more detail about the distribution of suicidal behaviour by gender and anger, see Figs. 1 and 2, respectively.

Factors Associated with suicidal behavior among Adolescent participant

In bi-variable logistic regression Age, sex, living status, ever use of alcohol, family history of suicidal attempts, growing up with a parent, relationship with a parent, social support, adverse childhood experience, stressful life experience, anger out expression behaviour, depression, interpersonal stress, and current use of tobacco were candidates for multiple variable regression at $p < 0.2$. In the multivariate analyses, family history of suicidal attempts, facing stressful life experiences, having adverse childhood experiences, having poor social support, not having a

good relationship with their parents, depression, interpersonal stress, and having high anger expression behaviours were found to be significantly associated with suicidal behaviour at $p < 0.05$.

Adolescents with a family history of suicidal attempts had a 5.66 times (AOR = 5.66, CI = 2.44, 13.10) higher risk of having suicidal behaviour compared to those who had no family history of suicidal attempts. Similarly, adolescents with stressful life experiences had higher odds (AOR = 2.91, CI = 1.45–4.91) of having suicidal behaviour compared to their counterparts. Adolescents with adverse childhood experiences (abuse, neglect, and household dysfunction) had a sevenfold (AOR = 7.11, CI = 3.56–14.20) higher risk of having suicidal behaviour compared to their counterparts.

Participants who lacked a good relationship with their parents had higher odds (AOR = 5.59, CI = 2.59–12.08) of developing suicidal behaviour compared to those who had a good relationship with their parents. Participants who had high interpersonal stress had approximately twice the odds [AOR = 2.17, 95% CI = 1.07.4.40] of suicidal behaviour compared to those who had low interpersonal stress.

Adolescents who had higher anger expression behaviour had 7.3 times the odds of reporting suicidal behaviour (AOR = 7.30, CI = 2.51-21.22) than those who had lower anger expression behaviour. Those who had depression had approximately five times the odds [AOR = 5.32, CI = 2.64–10.70] of suicidal behaviour. Adolescents who had poor social support were about 12.42 times [AOR = 12.42, CI = 5.95–25.95] more likely to report suicidal behaviour than participants who had good social support (table 3).

Discussion

The main objective of this study was to assess the prevalence and associated factors of suicidal behaviour among adolescents in Bahir Dar city. The prevalence of suicidal behaviour was 19.8% (125) (95% CI: 16.6-22.8) and the magnitude was different among males and females [males = 12.72%, females = 25.57%]. This finding was consistent with a study conducted in Ethiopia among adolescent high school students in Dangila Town, which showed that 16.2% had attempted suicide and 22.5% had suicidal ideation(23). The finding of this study was also consistent with another study conducted in Ghana among adolescent high school students who

reported suicidal behaviours: 18.2%, 22.5%, and 22.2% for suicidal ideation, suicidal plan, and suicidal attempt, respectively(32);Mozambique (17.7%suicidal ideation and 18.5% suicidal attempt) (33)and Togo (16.5% suicidal thought) (34).

The finding in this study was higher than school-based study done in Kut City, reported suicidal behavior to be 8.3% (9) and Tunisia (9.6% suicidal ideation and 7.3% suicidal attempt) (35). In the contrary, the finding was lower than studies done in Liberia where prevalence of suicidal attempt and suicidal ideation were 33.7% and 26.8% among adolescent students respectively (36), Lebanese 28.9% suicidal ideation(1), Benin (23.2% suicidal thought and 28.3% suicidal attempt) (6). Perhaps, the discrepancy might be due to difference in socio-demographic, economic and cultural characteristics of respondents, sample size, study setting, and might also be due to difference in measurement tools used mini-international neuropsychiatric interview (MINI) Suicidal Scale was used in this study (24).

In the multivariate analyses, several factors were found to be significantly associated with suicidal behavior. Having a family history of suicidal attempts, facing stressful life experiences, having adverse childhood experiences, poor social support, not good relationship with their parent, having depression, and having high anger out expression behavior is significantly associated with suicidal behavior.

This study found that the odds of suicidal behavior were 12.42 times higher among adolescents who had poor social support compared to those who had good social support [AOR=12.42,CI=5.95,25.95]. Previous studies (5, 23, 37), also have found poor social support is positively associated with suicidal behavior. Adolescent who had no good relation with a parent had 5.59 times higher risk for suicidal behavior than those who had a good relationship with their parent [AOR=5.59, 95% CI (2.59, 12.08)]. the finding of the study was supported by the previous study done in china and Japan (17, 38).

Adolescents who had a history of suicidal behavior in their family tried to end their life 5.66 times more likely than adolescents having no family history of suicidal behavior [AOR=5.66, 95% CI (2.44, 13.10)] and no significant associations were observed between suicidal attempt and suicidal ideation and family history of suicidal behavior in the study conducted in dangila town (23) but other studies reported that, there were significant associations (37). The Possible

reason for the discrepancy might be the coping mechanism of the study subjects, and probably the sample size used and the study setting they used institutional-based study.

Participants who had high interpersonal stress had approximately 2 times the odds [AOR=2.17, 95% CI (1.07.4.40)] of suicidal behavior compared to those who had low interpersonal stress. Previous study also support that high interpersonal stress was significantly associated with suicidal behavior (39).The possible reason for this might be Stress in interpersonal domains, meaning stress that occurs in the context of relationships with others, has consistently been identified as a significant correlate or predictor of suicidal behavior in adolescence.

Participants who had depression had approximately five times the odds [AOR=5.32, 95% CI=2.64, 10.70) of suicidal behavior than those who had no depression. Previous studies (9, 22, 40), also have found adolescent had depression is positively associated with suicidal behaviors. Adolescent having adverse child-hood experience (abuse, neglect and household dysfunction) had seven fold (AOR=7.11, CI= 3.56-14.20) higher risk of having suicidal behavior compared to their counterparts previous studies also found that adolescent those experiencing abuse, physically hurt and experiencing violence is positively associated with suicidal behavior (27).

Participants who had to experiencing stressful life events 2.91 times the odds of suicidal behavior than their counterparts [(AOR= 2.91,95% CI=1.43,5.90)]. This finding was supported by previous Studies in which suicidal behavior was significantly favored by stressful life events (35, 41).

Adolescents who had higher anger expression behavior had around 7.3 times the odds of reporting suicidal behavior (AOR=7.30, CI= 2.51-21.22) than those who had lower anger expression behavior. The possible reason might be Individuals with anger-related problems exhibit a range of cognitive and interpersonal deficits that have a deleterious effect on well-being and contribute to greater risk for suicidal behaviors, such as poor social problem-solving ability and poor social reasoning The results of this study provide support for the hypothesis that high levels of outward anger expressions are significantly associated with symptoms of depression and suicidal behavior.

Limitation of the study

This study is not without its limitations. Because it is cross-sectional due to that causal relationships cannot be established between independent factors and suicidal behaviors. Information bias may occur during data collection. This study did not account for the presence of psychiatric or psychological disorders other than depression, such as schizophrenia, which has been associated with suicidal behavior. This study was conducted in one city in Ethiopia, which limits its generalizability to other settings.

Conclusion

The prevalence of suicidal behavior was relatively high in this study showing a significant public health concern among adolescents in Bahir Dar city that requires a great emphasis. No social support, depression, having family history of suicide, not good relationship with their parent, adverse childhood experience (emotional, physical abuse and house hold dysfunction) and experiencing stressful live events were significant with suicidal behavior. Therefore, the information obtained could make a meaningful contribution to suicide prevention program either at community level or at institutional level.

Recommendation

For Bahir Dar city administration

- To work collaboratively with mental health professionals to strengthen provision of education for all community related to social support and provide information on suicidal behavior and its intervention.

For family

- Listen carefully and talk openly with your children about their thoughts and feelings (specifically about suicide and/or self-harm) and make good relationship with them.

For health professionals

- Health care providers should spent time with close relatives of adolescents to let them know the importance of social support and maintaining good relationship with their child.

For Amhara Regional Health Bureau

- It is important raising awareness of the public about factors of suicidal behavior such as poor social support, having depression, having family history of suicide, poor relationship with their parent, adverse childhood experience and experiencing stressful live event through educational campaigns

For local policy makers

- To local policy makers in designing appropriate strategies for suicide prevention in this population.

For researcher

Finally, further research is required to see the causal association between factor in prospective design and to identify other possible associated factors which were not touched like other substances abuse (such as cannabis, cocaine, heroin, and amphetamine), temperament, and familial economic status

ABRIVATIONS AND ACRONYMS

ASSIST =Alcohol, Smoking, and Substance Involvement Screening Test version, ACE= Adverse childhood experience, AORs=Adjusted Odds Ratios, BSRS=Bergen Social Relationships Scale, MINI=Mini-international neuropsychiatric interview, PHQ-9=Patient Health Questionnaire-9, PI=Principal Investigator, RSES=Rosenberg Self-Esteem Scale, SLESQ=The Stressful Life Events Screening Questionnaire , SPSS=Statistical Package for Social Science, WH CIDI=World Mental Health Composite International Diagnostic Interview, WHO=World Health Organization

Acknowledgments

We would like to thank Bahir Dar University for providing ethical approval to conduct this research. Our deepest thanks go to all study participants, data collectors, and supervisors who spent their valuable time for the good outcome of the research work

Authors' contribution: Belete Birhan, Dr. Michael Beka, Habte Belete, Abdulbasit Sherfa, Gidey Rtbey, conceived and designed the study, developed data collection instruments, participated in the testing and finalization of the data collection instruments, supervised data collection and coordinated the study progress. Habte Belete and Abdulbasit Sherfa reviewed and edited the manuscript. Belete Birhan performed the statistical analysis and wrote all versions of the manuscript. All authors read and approved the final manuscript

Declaration

Ethics approval and consent to participate

The study was approved by the Institutional review board (IRB) of Bahir Dar University, College of Medicine and Health Science ***[REF.178/2021].*** Information about the study was explained to each study participant in the information sheet. Written informed consent was obtained from Adolescents aged 18 years and above (participants under the age of 18 were given verbal assent and written informed consent was obtained from their parents or guardians on their behalf).

Competing interests: The authors declare that there was no competing interest

Reference

1. Pengpid S, Peltzer K. Suicide attempt and associated factors among adolescents in five Southeast Asian countries in 2015. Crisis: The Journal of Crisis Intervention and Suicide Prevention. 2020.

2. World Health Organization. Adolescent mental health 28 September 2020 [

3. Sousa CMdS, Mascarenhas MDM, Gomes KRO, Rodrigues MTP, Miranda CES, Frota KdMG. Suicidal ideation and associated factors among high school adolescents. Revista de saude publica. 2020;54:33.

4. Zarrouq B, Bendaou B, Elkinany S, Rammouz I, Aalouane R, Lyoussi B, et al. Suicidal behaviors among Moroccan school students: prevalence and association with socio-demographic characteristics and psychoactive substances use: a cross-sectional study. BMC Psychiatry. 2015;15(1):284.
5. Abdu Z, Hajure M, Desalegn D. Suicidal behavior and associated factors among students in Mettu University, South West Ethiopia, 2019: an institutional based cross-sectional study. Psychology research and behavior management. 2020;13:233.
6. Randall JR, Doku D, Wilson ML, Peltzer K. Suicidal behaviour and related risk factors among school-aged youth in the Republic of Benin. PLoS one. 2014;9(2):e88233.
7. world health organaization. Preventing suicide: A global imperative. 2014.
8. Hollinger JM. Adolescent Attitudes Toward and Perceptions of Suicide, Stigma, and Help-Seeking Behavior. 2016.
9. Ismail IS, Abdulsatar K, Baey HA. Prevalence and associated factors of suicidal behavior among secondary school students in Kut City. Medical Journal of Babylon. 2011;8(4):561-8.
10. McKinnon B, Gariépy G, Sentenac M, Elgar FJ. Adolescent suicidal behaviours in 32 low-and middle-income countries. Bulletin of the World Health Organization. 2016;94(5):340.
11. Thornton VJ, Asanbe CB, Denton EgD. Clinical risk factors among youth at high risk for suicide in South Africa and Guyana. Depression and anxiety. 2019;36(5):423-32.
12. Bifftu BB, Dachew BA, Tiruneh BT, Guracho YD. Prevalence of suicidal ideation, suicidal attempt and completed suicide in Ethiopia: a systematic review and meta-analysis protocol. Systematic Reviews. 2019;8(1):72.
13. Mars B, Burrows S, Hjelmeland H, Gunnell D. Suicidal behaviour across the African continent: a review of the literature. BMC Public Health. 2014;14(1):606.
14. World Health Organization. Live life: preventing suicide. 2018.
15. McMahon EM, Keeley H, Cannon M, Arensman E, Perry IJ, Clarke M, et al. The iceberg of suicide and self-harm in Irish adolescents: a population-based study. Social psychiatry and psychiatric epidemiology. 2014;49(12):1929-35.
16. Lim K-S, Wong CH, McIntyre RS, Wang J, Zhang Z, Tran BX, et al. Global lifetime and 12-month prevalence of suicidal behavior, deliberate self-harm and non-suicidal self-injury in children and adolescents between 1989 and 2018: a meta-analysis. International journal of environmental research and public health. 2019;16(22):4581.

17. Liu X, Chen H, Liu Z, Wang J, Jia C. Prevalence of suicidal behaviour and associated factors in a large sample of Chinese adolescents. Epidemiology and psychiatric sciences. 2019;28(3):280-9.
18. Strandheim A, Bjerkeset O, Gunnell D, Bjørnelv S, Holmen TL, Bentzen N. Risk factors for suicidal thoughts in adolescence-a prospective cohort study: the Young-HUNT study. BMJ open. 2014;4(8).
19. Rudatsikira E, Muula AS, Siziya S. Prevalence and associated factors of suicidal ideation among school-going adolescents in Guyana: results from a cross sectional study. Clinical Practice and Epidemiology in Mental Health. 2007;3(1):1-5.
20. Gvion Y, Apter A. Suicide and suicidal behavior. Public health reviews. 2012;34(2):9.
21. Liu Z-Z, Wang Z-Y, Bo Q-G, Qi Z-B, Xu R-J, Jia C-X, et al. Suicidal behaviours among Chinese adolescents exposed to suicide attempt or death. Epidemiology and Psychiatric Sciences. 2020;29.
22. Pandey AR, Bista B, Dhungana RR, Aryal KK, Chalise B, Dhimal M. Factors associated with suicidal ideation and suicidal attempts among adolescent students in Nepal: Findings from Global School-based Students Health Survey. PloS one. 2019;14(4):e0210383.
23. Amare T, Meseret Woldeyhannes S, Haile K, Yeneabat T. Prevalence and associated factors of suicide ideation and attempt among adolescent high school students in Dangila town, Northwest Ethiopia. Psychiatry journal. 2018;2018.
24. Roaldset JO, Linaker OM, Bjørkly S. Predictive validity of the MINI suicidal scale for self-harm in acute psychiatry: a prospective study of the first year after discharge. Archives of suicide research. 2012;16(4):287-302.
25. Kocalevent R-D, Berg L, Beutel ME, Hinz A, Zenger M, Härter M, et al. Social support in the general population: standardization of the Oslo social support scale (OSSS-3). BMC Psychology. 2018;6(1):31.
26. Hanlon C, Medhin G, Selamu M, Breuer E, Worku B, Hailemariam M, et al. Validity of brief screening questionnaires to detect depression in primary care in Ethiopia. Journal of affective disorders. 2015;186:32-9.
27. Murphy A, Steele M, Dube SR, Bate J, Bonuck K, Meissner P, et al. Adverse childhood experiences (ACEs) questionnaire and adult attachment interview (AAI): Implications for parent child relationships. Child abuse & neglect. 2014;38(2):224-33.

28. Rosenberg M. Rosenberg self-esteem scale (RSE). Acceptance and commitment therapy Measures package. 1965;61(52):18.

29. Terasaki DJ, Gelaye B, Berhane Y, Williams MA. Anger expression, violent behavior, and symptoms of depression among male college students in Ethiopia. BMC public health. 2009;9(1):1-8.

30. Green BL, Chung JY, Daroowalla A, Kaltman S, DeBenedictis C. Evaluating the cultural validity of the stressful life events screening questionnaire. Violence Against Women. 2006;12(12):1191-213.

31. Kopp MS, Thege BK, Balog P, Stauder A, Salavecz G, Rózsa S, et al. Measures of stress in epidemiological research. Journal of psychosomatic research. 2010;69(2):211-25.

32. Asante KO, Kugbey N, Osafo J, Quarshie EN-B, Sarfo JO. The prevalence and correlates of suicidal behaviours (ideation, plan and attempt) among adolescents in senior high schools in Ghana. SSM-population health. 2017;3:427-34.

33. Seidu A-A, Amu H, Dadzie LK, Amoah A, Ahinkorah BO, Ameyaw EK, et al. Suicidal behaviours among in-school adolescents in Mozambique: Cross-sectional evidence of the prevalence and predictors using the Global School-Based Health Survey data. PloS one. 2020;15(7):e0236448.

34. Darré T, Consuela KAC, Saka B, Djiwa T, Ekouévi KD, Napo-Koura G. Suicidal ideation and suicide attempts in subjects aged 15–19 in Lomé (Togo). BMC research notes. 2019;12(1):1-4.

35. Guedria-Tekari A, Missaoui S, Kalai W, Gaddour N, Gaha L. Suicidal ideation and suicide attempts among Tunisian adolescents: prevalence and associated factors. The Pan African Medical Journal. 2019;34.

36. Quarshie EN-B, Onyeaka HK, Asante KO. Suicidal behaviours among adolescents in Liberia. BMC psychiatry. 2020;20(1):1-12.

37. Giru BW. Prevalence and associated factors of suicidal ideation and attempt among high school adolescent students in Fitche Town, north Shoa, Oromia region, Ethiopia, 2012: Institutional based cross sectional study. Journal of Health, Medicine and Nursing. 2016;23.

38. Nagamitsu S, Mimaki M, Koyanagi K, Tokita N, Kobayashi Y, Hattori R, et al. Prevalence and Associated Factors of Suicidality in Japanese Adolescents: Results from a Population-based Questionnaire Survey. 2020.

39. Buitron V, Hill RM, Pettit JW, Green KL, Hatkevich C, Sharp C. Interpersonal stress and suicidal ideation in adolescence: An indirect association through perceived burdensomeness toward others. Journal of Affective Disorders. 2016;190:143-9.
40. Adewuya AO, Oladipo EO. Prevalence and associated factors for suicidal behaviours (ideation, planning, and attempt) among high school adolescents in Lagos, Nigeria. Eur Child Adolesc Psychiatry. 2020;29(11):1503-12.
41. Steele MM, Doey T. Suicidal behaviour in children and adolescents part 1: etiology and risk factors. Canadian journal of psychiatry. 2007;52(6):21S.

Table 1: Distribution of socio-demographic characteristics of adolescent participants in Bahir Dar city, Northwest Ethiopia, 2021 (n = 631).

**Variable**	**Category**	**Frequency**	**Percentage**
**Sex**	Female	348	55.2
	Male	283	44.8
**Age**	Early adolescent(10-13)	75	11.9
	Middle adolescent(14-17)	373	59.1
	Late adolescent(18-19)	183	29
**Marital status**	Single	627	99.4
	Married	2	0.3
	Divorced	2	0.3
**Religion**	Orthodox	509	80.67
	Muslim	102	16.16
	Protestant	20	3.17
**Living status**	With family	528	83.7
	Alone	62	9.8
	Other	41	6.5
**Educational level**	No formal education	2	0.3
	Primary school	299	47.4
	High school and above	330	52.3

*other (participants live with grandmother, grandfather, aunt, uncle)

*Table **2.** Frequency distribution of psychosocial and substance-related factors among adolescent participants in Bahir Dar city, Northwest Ethiopia, 2021 (n = 631).*

**Variable**	**Category**	**Frequency**	**Percentage**

Family history of suicide attempt	yes	85	13.5
	no	546	86.5
Family history of suicidal committed	yes	2	0.3
	no	629	99.7
Grow up with your biological family	yes	503	79.7
	no	128	20.3
Relationship with parent	good to very good	542	85.9
	not good/disturbed or very disturbed	89	14.1
Relationship with peers	good	629	99.7
	poor	2	0.3
Social support	poor	251	39.8
	moderate	300	47.5
	strong	80	12.7
Ever used tobacco/ cigarettes	yes	28	4.4
	no	603	95.6
In the past three month have you used tobacco/cigarettes	yes	23	3.6
	no	608	96.4
In your life, have you Ever used alcohol	yes	58	9.2
	no	573	90.8
in the past three month have you used alcohol	yes	50	7.9
	no	581	92.1
Ever use of khat	yes	32	5.1
	no	599	94.9
Khat use in the past three month	yes	26	4.1
	no	605	95.9

Table 3. The bi-variable and multivariable logistic regression analysis results of suicidal behaviour and associated factors among adolescent participants in Bahir Dar city, Northwest Ethiopia, 2021 (n = 631)

Variables	Category	Suicidal behavior		COR(95%Cl)	AOR(95%Cl)	p-value
		N (%) no	N (%) yes			
Sex	Male	247	36	1		
	Female	259	89	2.36(1.54,3.51)	1.52(0.75,3.07)	0.24
Age	Early adolescent (10-13)	64	11	1		
	Middle adolescent(14-17)	31 9	54	0.98(0.49,1.99)	0.69(0.23,2.04)	0.50
	Late adolescent(18-19)	12 3	60	2.83(1.39,5.77)	1.25(0.40,3.90)	0.69
Living status	With family	441	87	1		
	Alone	39	23	2.91(1.70, 5.26)	0.47(0.17,1.27)	0.14
	Other	26	15	2.92(1.49,5.75)	0.98(0.31,3.05)	0.90
Family history of suicidal attempt	No	463	83	1		
	Yes	43	42	5.45(3.35,8.85)	**5.66(2.44,13.10)**	**≤0.01****
Stressful life events experience	No	343	30	1		
	Yes	163	95	6.66(4.25,10.45)	**2.91(1.43,5.90)**	**≤0.01****
Adverse child-hood experience	No	383	19	1		
	Yes	123	106	17.37(10.24,29.48)	**7.11(3.56,14.20)**	**≤0.01****
Relationship with parent	Good to very good	465	77	1		
	Disturbed or very disturbed	41	48	7.07(4.36,11.44)	**5.59(2.59,12.08)**	**≤0.01****
Grow up with biological parent	Yes	443	60	1		
	No	63	65	7.62(4.91,11.82)	1.41(0.61,3.21)	0.41
Anger out	Low anger	134	16	1		

expression	expression					
	Moderate anger expression	327	67	1.72(0.96,3.06)	1.77(0.74,4.26)	0.19
	High anger expression	45	42	7.82(4.01,15.23)	**7.30(2.51,21.22)**	**≤0.01****
Social support	Good social support	364	16	1		
	poor social support	142	109	17.46(9.98,30.55)	**12.42(5.95,25.95)**	**≤0.01****
Depression	No	443	67	1		
	Yes	63	58	6.09(3.92,9.44)	**5.32(2.64,10.7)**	**≤0.01****
Ever use of alcohol	No	476	97	1		
	Yes	30	28	4.59(2.62,8.01)	1.50(0.53,4.20)	0.44
Current use of tobacco	No	494	114	1		
	Yes	12	11	3.97(1.71,9.23)	0.26(0.59,1.14)	0.75
Interpersonal stress	Low stress high stress	273<br>233	33<br>92	3.26(2.11, 5.04)<br>1	**2.17(1.07.4.40)**	**0.03**

*P value is significant at P<0.05 ** P value is significant at P<0.01 P value of Hosmer and Lemeshow Test=0.939

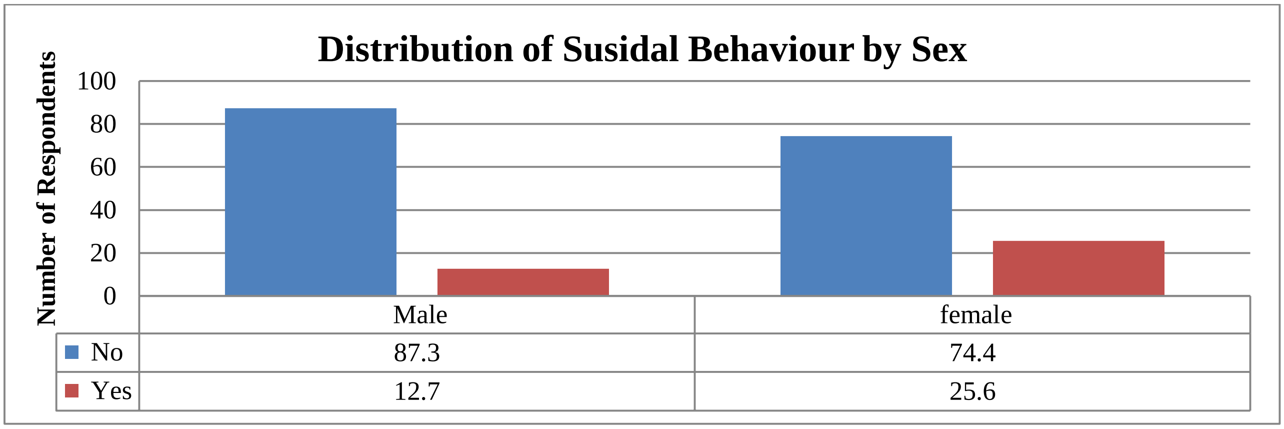

Figure 1: Distribution of suicidal behaviour by gender among adolescents in Bahir Dar city, Northwest Ethiopia, 2021

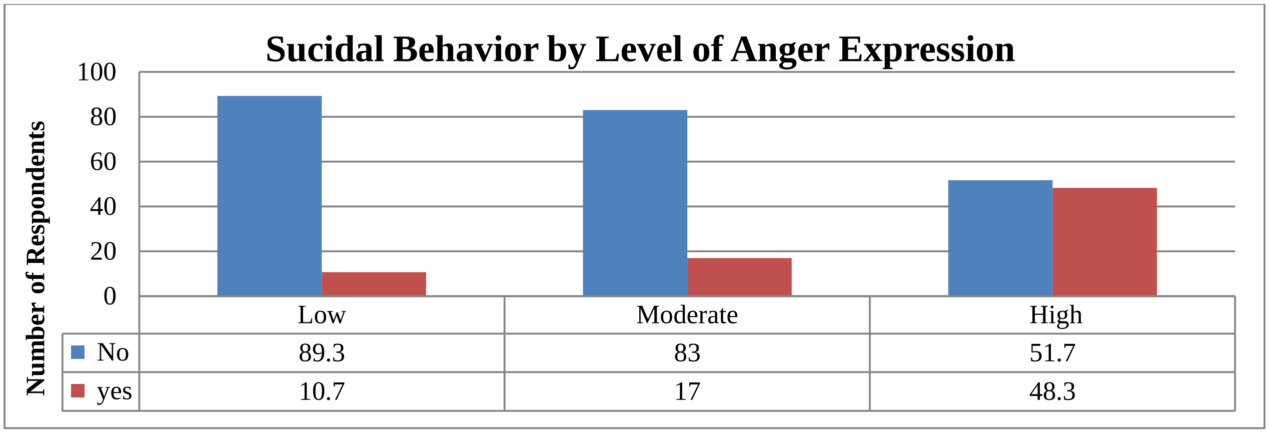

Figure 2: Distribution of suicidal behaviour by level of anger expression among adolescents in Bahir Dar city, Northwest Ethiopia, 2021
