# Supplementary material for: Magnitude and associated factors of sexual violence among female housemaids attending night school in Bahir Dar City, Northwest Ethiopia: Institution-Based Cross-Sectional Study, 2022
Source: PLoS One. 2024 Jul 25;19(7):e0307308. doi: 10.1371/journal.pone.0307308 (PMC11271934; doi:10.1371/journal.pone.0307308)
Supplement: Attachment — Submitted filename: Reviewer Evaluation.docx [file pone.0307308.s001.docx]

**Reviewer Evaluation**

**Title:** **Magnitude and associated factors of sexual violence among female housemaids attending night school in Bahir Dar City, North West Ethiopia.**

Thank you for inviting to review this manuscript.

-When just starting from the title add study design and year of study.

**Abstract**

**-**Remove the abbreviation from the abstract

-Merge objective and background

- Ever had sex was significantly associated with sexual violence after being housemaids. How it could be related to lifetime sexual experience with being housemaids

-All variables are not self-explanatory For example: is social support low or strong?

**-Conclusion:** The magnitude of sexual violence among housemaid has been found to be high
compare to previous findings. Its seems comparative study. Be fixed with your topic. Needs revision.

**Introduction:** Most of the introduction section deals only with violence among women in the general population rather than housemaids. Also very wide please fix with your topic.

-**Data collection procedure:** I disagreed with the tool, it has no clear-cut point and complete or standard questionnaire. Please attach it.

-Where pretest was conducted?

- Why do you prefer P -value less than 0.25 for a multi-variable logistic regression analysis?

- Hosmer-Lemeshow goodness test was fitted to check model fitness. 0.19. Remove the point after the word fitness.

**Result:**

-Please separate the frequency and percentage of each variable with a column.

-How did you categorize each variable like age, level of grade and etc?

-How did you see the difference between family support and social support? No one of the participants had strong social support.

- Statistical analysis has not been performed appropriately. Some of the cross-tabulation (ad/bc) are written inversely and check revised correctly.

-Add P-Value for each variable in multivariable regression analysis.

Discussion:

-Poor discussion compared with other studies. Revise it again.

-Hong Kong 25.38%, remove column before the reference.

-Be consistent in using capital letters and punctuation. It has also several grammatical errors. Please show for native speakers.

- The result of your study is in line with others no need for discussion. This implies that the burden of sexual violence is more prevalent throughout the world. Despite the human right approach, everyone has the right to control their own body group of population(47). Remove it.

-The reason for the variation in magnitude needs further explanation.

-Add implication of your study

-Conclusions; add another conclusion.

-Verbal consent was used why not written?

- Please attach a copy of the Ethical approval letter.

- Update all references older than 10 years to a more recent study.

-General Comments: the grammar needs attention throughout the document, especially the introduction, results, and discussion sections.

------------In general even though this study is relevant it has several grammatical errors, an unattractive written introduction. I do not agree to publish at this stage. The authors expect to revise exhaustively by incorporating all the above issues.

Best regard,
