# Supplementary material for: Magnitude and associated factors of sexual violence among female housemaids attending night school in Bahir Dar City, Northwest Ethiopia: Institution-Based Cross-Sectional Study, 2022
Source: PLoS One. 2024 Jul 25;19(7):e0307308. doi: 10.1371/journal.pone.0307308 (PMC11271934; doi:10.1371/journal.pone.0307308)
Supplement: Attachment — Submitted filename: Response to reviwers.docx [file pone.0307308.s002.docx]

Point by point response

|  | **Reviewers comment** |  |
| --- | --- | --- |
|  |  | **Authors response** |
|  | Reviewer 1: |  |
| 1. | The document is poorly written grammatically. I didn't understand some parts of the document. eg. under conclusion it says Therefore 'resolve this problem' ?? so what? this phrase lacks sense in your sentence. | Thank you for your comment. To address the language issue, we consulted senior public health staff and language professors at my university. We also used online software, specifically Grammarly and scribens (to correct grammar and spelling), throughout the manuscript.  In addition, Thank you for your comment. We accept rewrite this section as follows: “Therefore, creating a sexual and reproductive health (SRH) discussion session for housemaids is important for securing their sexual rights.” in the updated manuscript. |
| 2. | The conclusion part in abstract is broader than and different from the one written in the main document. Please revise them | Thank you for your comment. We accept and update the conclusion section in the updated manuscript.  Line numbers 51 to 52 |
| 3. | Sometimes you use abbreviation eg SRH which not internationally known and its meaning also varies in the document. so please make it similar either sexual and reproductive health or sexual and reproductive issue. In addition the term discussion can better be clear if you revise or add some term at a from like having discussion of sexual and reproductive health with family or having talk about sexual and reproductive health with partner pla..pla.. because the variable is vage. | Thank you for your comment. We accept and define the abbreviation in the update manuscript. Discussion with sexual and reproductive health was measure whether the housemaids discuss any one( friends, partners, family members, employer and teachers) in the past twelve months and we revise it in the updated manuscript. |
| 4. | One variable is also reported as time to school but under introduction the discussed factor is distance from school so please clearly justify under discussion or try to manage the variation. | Thank you for your constructive comments. We measure distance from school with walking hours. Based on your comment we consistently used distance instead of time to school so we used distance to school in the updated manuscript. |
| 5. | Under introduction the name of country and states are mixed and some reports lack evidence eg. It reports Brazil as a developed nation? Considers Texas as a country? | Thank you for your comment. based on your comment we revised it as follows: “Studies conducted in the globe showed that the magnitude of sexual violence among female housemaids ranges from 14% to 29%” in the updated manuscript.  Line numbers 88 to 89 |
| 6. | Under methods and materials section around line 8 please add study design and also write the study period in a similar way with the one found under Abstract section. | Thank you for your constructive comments. We amended all the comments in the updated manuscript.  Line numbers 88 to 89 |
| 7. | Please specify the clear number of BSc Midwifes and Psychiatry nurse involved in your study? | Thank you for your constructive comments. based on your comment we separately specify numbers of BSc Midwifes and Psychiatry nurse in the updated manuscript.  Line numbers 152 to 153 |
| 8. | I didnt like the way you wrote your result. You sometimes write in word and otherwise in figure it seems searching for way out from plagiarism. better to write both frequency and percentage in figures and specific to some findings from the table. | Thank you for your constructive comments. We amended all the comments in the updated manuscript. |
| 9. | Summation of the percentages in the table is above 100. eg.for religion and marital status. | Thank you for your constructive comments. We amended all the comments in the updated manuscript.  Table 1 |
| 10. | Under discussion please revise the name of countries or cities you used for discussion purpose and add more strong justifications. | Thank you for the suggestion. Based on your comment, we revised the whole discussion section in the updated manuscript.  Line numbers 228 to 273 |
|  | Reviewer 2 |  |
| 1. | **Abstract**  It is good but the structure and spelling errors; | Thank you for your comment. To address the structural and spelling issue, we consulted language professors at my university and online software, specifically Grammarly and scribens (to correct grammar and spelling), throughout the manuscript. |
|  | In the conclusion section of the abstract the factors should be written clearly for readers for example those who have poor school performance etc., | Thank you for the suggestion. Based on your comment, we clarify the variable in the updated manuscript.  Line number 54 |
|  | The recommendation in the conclusion section is not specific and based on the pertinent finding. | Thank you for your constructive comments. We revised the recommendation section in the updated manuscript.  Line numbers 55 to 56 |
| 2. | Introduction; Grammar, punctuation, and the consequences of sexual violence are not briefed | Thank you for your comment. To address the language issue, we consulted senior public health staff and language professors at my university. We also used online software, specifically Grammarly and scribens (to correct grammar and spelling errors), throughout the manuscript.  In addition, we tried to highlight the consequences of sexual violence in the updated manuscript.  Line number 63 to 66 |
| 3. | Method;  Separate the study area and study design separately, with a population (source and study population) as a subtitle. | Thank you for your constructive comments. We incorporate all your concern in the updated manuscript.  Line number 118 to 123 |
|  | An operational definition should be clear (discussed briefly the items in short) and it should not be written all in one paragraph (one paragraph for one variable),  specifying where the pretest was done. | Thank you for your constructive comments. We incorporate all your concern in the updated manuscript.  Line number 136 to 149  The pretest was held in Debre Tabor Town, about 100 kilometers north of Bahir Dar. |
|  | Data analysis section lines 6-8 is too long and unclear for readers (rewrite it scientifically). | Thank you for your suggestion. We revised the suggested section in the updated manuscript.  Line number 166 to 168 |
|  | You do have an age of less than 20 participants means that there might be under 18 participants (checked SD±2.28), so how did you see the ethical concern of these participants? | Yes, there are participants under the age of 18. As a result, we obtain assent from guardians or parents for all participants under the age of 18.  Line number 175 to 176 |
| 4. | Result: Table 1; do you mean male partner, | Thank you for your suggestion. We wants to say male household member who use alcohol not male partner only. |
|  | the way that you measured the magnitude of sexual violence is not specifically briefed in the result section (you only discussed the general magnitude of sexual violence). | Thank you for your suggestion. We revised the suggested section in the updated manuscript.  Line number 204 to 209 |
|  | Check and take some corrections on the odds of ……the way that you discussed the factors (AOR). Table 3; avoid COR, you should include the %value of each variable, and indicate with Astrix (**) the associated factor in the final model. | Thank you for your suggestion. We revised the suggested section in the updated manuscript. |
|  | The way how did you measure the discussion about SRH should be clearly shown in the operational definition;  the variables that you use are somehow few; in the last section of table 3 what does the average score mean (Avoid it)? | Thank you for your constructive comments. We include how we measured SRH in the operational definition section.  Line number 150 to 153  Average score mean academic performance of the student. To make it clear, we change the average score by academic performance and we avoid it in table three |
| 5. | Discussion; Line one said that These studies identify…..[correct it]……respectively (need correction) When you discuss the magnitude you should modify the grammar, the way that you discussed, and the way that you justify should be clear (It is good but it is not written scientifically).  Discussion about factors must rewrite it as first your finding then the study which is consistent or inconsistent with your finding, reference, then possible justification (You should correct the factors section) | Thank you for the suggestion. Based on your comment, we revised the whole discussion section in the updated manuscript including the grammar.  Line numbers 234 to 281 |
|  | Conclusion; Is not addressed based on your pertinent finding and your objective Check the reference some of your references do not exist in the online system. | Thank you for the suggestion. We revised the conclusion section in the updated manuscript.  Line Number 282 to 289 |
|  | Reviewer 3 |  |
| 1. | When just starting from the title add study design and year of study. | Thank you for your constructive comments. We amend all the comments on the updated manuscript as follows: "institutional-based cross-sectional study, 2022."  Line numbers 2 to 3 |
| 2. | **Abstract**  **-**Remove the abbreviation from the abstract  -Merge objective and background | Thank you for your constructive comments. We amended all the comments in the updated manuscript.  Line numbers 26 to 50 |
|  | - Ever had sex was significantly associated with sexual violence after being housemaids. How it could be related to lifetime sexual experience with being housemaids | Thank you for your constructive comments. As you mentioned, having ever had sex was a significant variable, which means housemaids with a history of sex were more likely to experience sexual violence after becoming a housemaid, but does not mean that having ever had sex makes them a housemaid. |
|  | -All variables are not self-explanatory For example: is social support low or strong? | Thank you for your constructive comments. We amended all the comments by rename it as follows: "poor social support" in the updated manuscript.  Line number 45 to 46. |
| 3. | **Conclusion:** The magnitude of sexual violence among housemaid has been found to be high compare to previous findings. Its seems comparative study. Be fixed with your topic. Needs revision. | Thank you for your comment. We accept and update the conclusion section in the updated manuscript.  Line numbers 51 to 52 |
| 4. | Introduction  Most of the introduction section deals only with violence among women in the general population rather than housemaids. Also very wide please fix with your topic. | Thank you for the suggestion. Based on your comment, we accept and amend all the corrections in the updated manuscript.  Line numbers 60 to 108 |
| 5. | -**Data collection procedure:** I disagreed with the tool, it has no clear-cut point and complete or standard questionnaire. Please attach it. | Thank you for the suggestion. Actually, we used WHO standard violence measurement tools to assess whether housemaids were sexually violated or not. We used three yes/no questions, and if the housemaid answered yes to any of them, she was considered sexually abused. To make this more clear, we revised the operational definitions of the outcome variables as follows: “A WHO standard tool was used to assess sexual violence. Three independent Yes/No questions were asked for each housemaid. Whether or not their husband has ever physically forced them to have sex or engage in other sexual acts, they do not want to. Did your current husband/partner or any other partner ever physically force you to have sexual intercourse when you did not want to? Did you ever have sexual intercourse you did not want to have because you were afraid of what your partner or any other partner might do? Did your partner or any other partner ever force you to do something sexual that you found degrading or humiliating? If a housemaid answered yes to one of the three questions, she was considered sexually violated”  Line number 136 to 153 |
|  | -Where pretest was conducted? | The pretest was held in Debre Tabor Town, about 100 kilometers north of Bahir Dar. |
|  | - Why do you prefer P -value less than 0.25 for a multi-variable logistic regression analysis? | Thank you for your comment. We used a p-value less than 0.25 to deduce the numbers of variables for multivariable analysis, and we believe that those variables whose p values greater than 0.25 were less likely to be statistically significant in multivariable analysis. |
|  | - Hosmer-Lemeshow goodness test was fitted to check model fitness. 0.19. Remove the point after the word fitness. | Thank you for the suggestion. Based on your comment, we amended all the corrections in the updated manuscript. |
| 6. | **Result:**  -Please separate the frequency and percentage of each variable with a column. | Thank you for your suggestion. Based on your comment, we amended all the corrections in the updated manuscript. |
|  | -How did you categorize each variable like age, level of grade and etc? | Thank you for the comment. We use previous research and standards to categorize each variable. For example, we categorize age as less than 20 (ages 15 to 19) and greater than or equal to 20 (>=20). Similarly, we categorize educational level based on standard categories: primary (grades 1–8), secondary (grades 9–12), and similar for the rest of the variables. |
|  | -How did you see the difference between family support and social support? No one of the participants had strong social support. | Family support is the support of families, in which family members can assist by providing medical information, making time for emotional sharing. While social support is the perception and reality that one is cared for, there is also assistance available from other people, including friends, colleagues, and acquaintances.  We used a standard Oslo 3 social support measurement scale, but no participants scored good social support on it, so we simply classified it as moderate and poor. |
|  | Statistical analysis has not been performed appropriately. Some of the cross-tabulation (ad/bc) are written inversely and check revised correctly. | Thank you for your comment. I see the statistical analysis conducted rigorously, including the cross-tabulation, and it seems fine. In some of the cross tabulations, we may have used the last categories (response variables) as reference categories that seems reversed. |
|  | -Add P-Value for each variable in multivariable regression analysis.  ……" | Thank you for the suggestion. Based on your comment, we include  p-value in the updated manuscript.  Line numbers 223 |
|  | Discussion:  -Poor discussion compared with other studies. Revise it again. | Thank you for the suggestion. Based on your comment, we revised the whole discussion section in the updated manuscript.  Line numbers 234 to 281 |
|  | -Hong Kong 25.38%, remove column before the reference. | Thank you for the suggestion. Based on your comment, we amended the corrections in the updated manuscript. |
|  | -Be consistent in using capital letters and punctuation. It has also several grammatical errors. Please show for native speakers. | Thank you for your comment. To address the language issue, we consulted senior public health staff and language professors at my university. We also used online software, specifically Grammarly and scribens (which check for correct grammar and spelling), throughout the manuscript. |
|  | - The result of your study is in line with others no need for discussion. This implies that the burden of sexual violence is more prevalent throughout the world. Despite the human right approach, everyone has the right to control their own body group of population (47). Remove it. | Thank you for the suggestion. Based on your comment, we remove it in the updated manuscript. |
|  | -The reason for the variation in magnitude needs further explanation. | Thank you for your suggestion. We revised the entire manuscript in the updated version based on your feedback. |
|  | -Add implication of your study | Thank you for your suggestion. We include implication in the updated version based on your feedback. |
|  | -Conclusions; add another conclusion. | Thank you for the suggestion. We revised the conclusion section as follows: "This study identified that the magnitude of sexual violence among housemaids was high. Housemaids' sexual violence was found to be significantly associated with ever having sex, no discussion of sexual and reproductive health (SRH), poor social support, school performance, and time taken to get to school. Therefore, developing a strategy to increase sexual and reproductive health information for night school students is critical for reducing sexual violence. In addition, providing psychosocial support for the victim is important to reduce the risk of adverse psychosocial effects," in the updated manuscript.  Line Number 282 to 289 |
| **7.** | -Verbal consent was used why not written? | Thank you for your comment. Please accept my apologies for these careless editorial errors. Actually, we take written consent. We accept and amend the comment in the updated manuscript. |
|  | - Please attach a copy of the Ethical approval letter. | Thank you for the suggestion. We upload the ethical approval letter. |
|  | - Update all references older than 10 years to a more recent study. | Thank you for the suggestion. Based on your comment we accept and amend all the correction on the updated manuscript. |
|  | -General Comments: the grammar needs attention throughout the document, especially the introduction, results, and discussion sections. | Thank you for your comment. To address the language issue, we consulted senior public health staff and language professors at my university. We also used online software, specifically Grammarly and scribens (to correct grammar and spelling errors), throughout the manuscript. |
