# Supplementary material for: Magnitude and associated factors of sexual violence among female housemaids attending night school in Bahir Dar City, Northwest Ethiopia: Institution-Based Cross-Sectional Study, 2022
Source: PLoS One. 2024 Jul 25;19(7):e0307308. doi: 10.1371/journal.pone.0307308 (PMC11271934; doi:10.1371/journal.pone.0307308)
Supplement: Attachment — Submitted filename: Point-by-point response.docx [file pone.0307308.s004.docx]

| Point-by-point response | | |
| --- | --- | --- |
| We sincerely thank the reviewers for their thorough review of the manuscript and for providing constructive feedback. We believe we have thoroughly incorporated their comments into the updated version. | | |
| Reviewers Comment | Revised versions | |
| **Reviewer 1.** |  | |
| This manuscript needs more language editions. |  | |
| Why authors choose this time frame (May 15 to June 20)? | There was no specific reason for choosing May and June, but it aligned with our work schedule. Additionally, the academic calendar in Ethiopia runs from September to June. Therefore, we collected the data before the students left for vacation. Given the total number of eligible study participants, the sample size, and the sampling technique, approximately 35 days were sufficient for collecting data from the entire sample population. | |
| A simple random sampling, how it could be? or how did authors made internal and external validity? | This study included all sixteen schools, both private and government, that offer evening programs. Initially, a preliminary census was conducted at each school to identify students working as housemaids by recording their school names, job types, grade levels, class numbers, and sections as a sampling frame. Subsequently, participants were selected using a computer-generated method from the list of the student.  The authors aimed to maintain the internal validity of this research through the careful design of the data collection tool, ensuring appropriate measurements were used for each variable included in the study. Similarly, training was given for data collection and the process was monitored by trained supervisors. Additionally, to maintain the generalizability of the findings, the sample size was meticulously determined. | |
| How was the measurement tools were adopted from literature or is it valid? how? | The data measurement tool was developed by reviewing various literature and WHO standard tools. After its development, both content and face validity were assessed by subject matter experts. Additionally, the tool was pretested to check consistency in the local context. | |
| Introduction: better to start what general violence mean? background lakes idea flow from global to local. | Thank you for the constructive comment. Based on your suggestion we include the general violence definition in the revised manuscript.  *“Violence is the intentional use of physical force or power, whether threatened or actual, against another person, which results in or has a high likelihood of resulting in injury, death, or psychological harm. While the majority of violence survivors are girls and women, boys and men can also be victims.”* | |
| line 91-93: if those studies have been done what are the real significance's of your study? | Thank you for raising this issue. Although sexual violence and its determinates in Ethiopia have been studied to some extent, there remains a notable gap concerning night school students employed as housemaids. Previous studies have shown a wide range in reported prevalence rates of violence (27.8% to 72%), indicating a need for further investigation to reconcile these discrepancies. Therefore, this study aims to contribute recent evidence on sexual violence among housemaids who attend night school, aiming to inform targeted local and national interventions for this overlooked segment of population. | |
| Line 101: how about Ethiopian school health program and violence? | Thank you for the constructive comment. Ethiopia's Growth and Transformation Plan II prioritizes addressing violence as a key focus area. However, the school health programs in Ethiopia are not well functioning. Consequently, essential services related to addressing violence are unavailable in the majority of schools across the country. | |
| Background (literatures )haven't told us about school and violence? | Thank you for the constructive comment. Based on your suggestion we revised the whole background section. | |
| line 116 and 117: how did Authors arrange for simple random sampling for both private and public schools? | Thank you for the constructive comment. This study encompassed all sixteen schools, both private and government, that offer evening programs. Initially, a preliminary census was conducted at each school to identify students working as housemaids by recording their school names, job types, grade levels, class numbers, and sections. Each eligible respondent was then assigned a unique code for identification. Participants were selected using a simple random sampling technique, utilizing computer-generated methods. | |
| line 124: Lacks figurative descriptions. How the private school could be represented? | Thank you for your valuable feedback. We did not employ proportional allocation for private and government schools because we did not consider schools as a variable, given that there is no difference between governmental and private night schools in Ethiopia, as all night school students are supposed to pay. Additionally, no school-level difference makes them vulnerable to violence. Therefore, we employed simple random sampling, to ensure that each student had an equal chance of being represented. | |
| For n, this study have done community based and your study is institutional at school how it could be a p value? | Thank you for your valuable feedback. Since there were few institutional-based studies conducted near our study area, we determined the sample size based on proportions derived from community-based studies. | |
| line 132 and 133: better to place in annex/appendix by securing privacy? | Thank you for your valuable feedback. Individual identifiers such as names and household numbers/addresses were not collected to maintain ethical standards. However, for sampling purposes during the preliminary census, data such as school names, job types, grade levels, class numbers, and sections were recorded. This approach ensured that their privacy and confidentiality were not compromised. | |
| School mean? primary or secondary or and tertiary please see your topic and amend it. | Thank you for your input. In this study, “school” refers to both primary and secondary education institutes, which is the common definition in the country. Whereas tertiary education is considered as college or university. Therefore, our study only considered schools (primary or secondary). | |
| line 137: operational definition need rewriting. | Thank you for your valuable feedback. Based on your comment we revised the operational definition in the revised manuscript.  ***“Social support****: Social support was measured using the Oslo 3 Social Support Scale, which consists of three questions with a total possible score of 14 points. Participants scoring 3–8 were categorized as having poor social support, those scoring 9–11 were categorized as having moderate social support, and those scoring 12–14 were categorized as having good social support.”* | |
| line 146: is it real validate for our context? evidence? How it could be in age difference? primary school vs secondary school age group? | Thank you for your input. We did not conduct a stratification analysis based on age and educational attainment. However, it's worth noting that most participants were around 18 years old, as indicated by the mean age of 18 years with a standard deviation of 2.28. | |
| line 150: how it could be for age less than 12 years? | Thank you for your comment. In our study, we did not observe any participants below 12 years of age. | |
| one MPH holder then supervisors. is it grammatically correct or make it clear? | Thank you for you reside the point. We corrected the error in the revised manuscript  *“ Two midwives with BSc degrees and two psychiatry nurses with BSc degrees were assigned as data collectors, with one supervisor holding a Master of Public Health (MPH)”* | |
| line 168 and 169: how it could be for all variables mean? and line 175: please provide a tangible value. | Thank you for you reside the point. We included the real value in the revised manuscript.  *“Descriptive statistics were computed to see the distributions of covariates.”*  *“The Hosmer-Lemeshow goodness test was fitted to check model fitness, which was found to be 0.65.”* | |
| line 180: how it could be for females age less than 18 years? line 176: What innervations were done by data collectors or investigators for those who have faced violence? | Thank you for your valuable comment. This study included both primary and secondary night school students, including those under 18 years old for whom parental consent was obtained. Participants who reported experiencing sexual violence were referred to community health agents for appropriate support and intervention. | |
| line 181: which informed consent? your data collection sites were schools? how did you get parents/guardians? | Thank you for your feedback. Before conducting the interviews, the data collectors made efforts to contact the parents of participants under 18 years old to obtain consent through written letters. | |
| line 183 and 184: what does it mean? needs grammatical correction. | Thank you for you reside the point. We corrected the grammatical error in the revised manuscript.  *“Furthermore, all participants were informed of their right to withdraw from the study at any point.”* | |
| table 1: the study were done in Bahir Dar city? what is the importance of reporting urban and rural? | Thank you for your feedback. The figure shows the place where the housemaids were born. It is not that important for current sexual violence. Therefore, we removed it from the revised manuscript. | |
| please see under 23- years and marital status plus how it could be only into categories? all variables in the table have confusion. | Thank you for your comment. We did not have under 23 age categories. We reported two marital status categories due to zero values for other categories such as widowed and married, which are typically omitted from tables. | |
| line 229 and 230: better to describe by percentage rather than inversion of odds ratio. | Thank you for you reside the point. Based on your suggestion we revised it in the updated manuscript | |
| line 284: base line to report high? and 293: What does it mean did you have evidence (at least up to your sample size)? | Thank you for your suggestion. We revised it by including *“Compared to similar literature”* in the revised manuscript. | |
| 293: What does it mean did you have evidence (at least up to your sample size)? | Thank you for the feedback. During data collection, we obtained both consent and assent, including consent for publication. We omitted this section as it is not required by the journal guidelines. | |
| **Reviewer 2.** | | |
| Except some fragmented sentence | Thank you for the feedback. We revised the entire document. | |
| Some portions in the document needs clarification and rewritten |  |  |
| The introduction part needs to be rewritten again by keeping logical sequence |  |  |
| English language improvement is needed in the whole document. | We revised the language by the senior researcher in the university and used online Grammarly. | |
|  | |  |
| The data for the total population of Bahir Dar City is not Up to date, please update it accordingly. | Thank you for your feedback. However, we don’t have any section which talks about Bahir Dar city's total population. | |
| How do you identify the adolescents living 6 month in the city? | Thank you for your feedback. We identify those residents through Self-report. | |
| How can you justify weather some sub cities have a more administrative kebeles than others comparably, How could you make it proportional. | Thank you for your comment. We utilized a simple random sampling technique by identifying lists of students from the schools. As a result, we did not employ proportional allocation in our sampling approach | |
| What was the K value for SRS bases household sampling. | Thank you for your feedback. Participants were selected using a simple random sampling technique with computer-generated methods. Since the authors did not use systematic random sampling, there was no need for a sampling interval (K^th^ interval). | |
| It would be better if you summarize by drawing the sampling tree in short and precise way? | Thank you for your feedback. A schematic presentation of the sampling procedure doesn’t seem suitable here. As mentioned in the third response, a preliminary census was conducted, followed by coding, and finally, a simple random sampling technique was employed. | |
| Is the pretest done for this study and how was the result of Cronbach alpha value to validate the tool? | A pretest was conducted among 10% (34 participants) of the participants to check the consistency and appropriateness of the tool outside of the study area. | |
| How do you evaluate the presence of other psychiatric condition like MDD and Schizophrenia and Epileptic conditions as it may contribute more to the suicidal attempt?  **Response:** this study included survivors. | Thank you for your feedback. For this study we did not assess any psychiatric disorders as awhole. | |
| Have you done any crosscheck in some samples through the other group who are responsible for the data quality to minimize bias by the data collectors? | Thank you for your feedback. Thorough training was given to the data collectors regarding the objective the study, the data collection tool and related issues. | |
| The data availability statement is not mentioned here? | Thank you for your comment. We revised the section in the revised manuscript. | |
